# *LACK* Gene’s Immune Response Induced by Cocktail DNA Vaccine with *IL-12* Gene Against Cutaneous Leishmaniasis in BALB/c Mice

**Published:** 2018

**Authors:** Oghlniaz Jorjani, Fatemeh Ghaffarifar, Zohreh Sharifi, Abdolhossein Dalimi, Hajar Ziaei-Hezarjaribi, Benyamin Talebi

**Affiliations:** 1. Laboratory Science Research Center, Golestan University of Medical Sciences, Gorgan, Iran; 2. Department of Parasitology, Faculty of Medical Sciences, Tarbiat Modares University, Tehran, Iran; 3. Research Center of Iranian Blood Transfusion Organizations, Tehran, Iran; 4. Department of Parasitology, Faculty of Medicine, Mazandaran University of Medical Sciences, Sari, Iran; 5. Private Veterinary Physician, Gorgan, Iran

**Keywords:** Cutaneous leishmaniasis, DNA vaccine, Immune response

## Abstract

**Background:**

Leishmaniasis is caused by parasitic protozoa of the genus *Leishmania* which is an obligate intracellular parasite in the infected host. Individuals who have been recovered from clinical leishmaniasis develop strong immunity against reinfection. DNA vaccines are the new type of vaccines that induce expression of protein eukaryotic cells. DNA vaccines can be stimulated by the cellular and humoral immune responses using one or several genes.

**Methods:**

A DNA vaccine containing plasmids encoding the *pcLACK+pcTSA* genes of *Leishmania major (L. major)* (MHRO/IR/75/ER) in the vicinity of *IL-12* gene expression was made and then its protective efficacy in comparison with single-gene of LACK was evaluated. Also, BALB/c mice were immunized intramuscularly three times. The humoral and cellular immune responses were evaluated after immunization with pcLACK, pcLACK+pcTSA+pCAGGS-IL12, and then challenged with *L. major.*

**Results:**

Humoral response and IFN-γ values were significantly higher than control groups after immunization with pcLACK, pcLACK+pcTSA+pCAGGS-IL12 and challenge with *L. major* (p≤0.05). IL-4 values were increased in the control groups in such a way that they were remarkably higher than the pcLACK, pcLACK+pcTSA+ pCAGGS-IL12 groups (p≤0.05) after immunization and challenge with *L. major*.

**Conclusion:**

The survival time of the immunized mice with pcLACK, pcLACK+pcTSA+ pCAGGS-IL12 groups was higher than the control groups. Then, DNA vaccine of pcLACK appeared to be likely able to induce more protection against infection with *L. major* in mice. Therefore, cocktail DNA is effective to enhance specific immunity.

## Introduction

Leishmaniasis is caused by a protozoan called *Leishmania major* (*L. major)* which is widespread throughout the world 1. Species of *Leishmania* cause human diseases that form cutaneous lesions to Visceral Leishmaniasis (VL). Additionally, Cutaneous Leishmaniasis (CL) is produced by *L. major*, *Leishmania tropica* (*L. tropica)* and *Leishmania aethiopica* (*L. aethiopica)* in the Old World 2,3. Almost 350 million people are at the risk of acquiring the various forms of the disease and also the annual incidence of new cases is around 1.5 million CL cases 4. Lack of effective, low-cost treatments and the irreversibility of tissue damage during infection make it necessary to develop an effective vaccine that is protective against diseases caused by diverse species of *Leishmania* 5.

Vaccination strategies are based on the current understanding of the characteristics of an effective anti-leishmania immune response. Studies on animal models emphasized the role of cellular immunity in controlling leishmaniasis 6.

Recently, DNA vaccine was considered as an effective method to control disease depending cellular immune response 7. In order to produce the DNA vaccine, preferred gene consists of a eukaryotic expression vector to encode antigen 8. In recent years, various antigens, *L. major,* were investigated as candidates for DNA vaccine. Antigens of leishmania homolog of receptors for activated c-kinase (LACK) and Thiol-Specific-Antioxidant (TSA) have been reported as the preventive vaccine candidates 9,10.

LACK is a 36 *kDa* protein and expressed in both stages of the leishmania parasite life cycle 11. *LACK* gene is placed on the chromosome 28 and is composed of 939 *bp* 12. This protein has been shown to be protective when used as a DNA vaccine in mice by redirecting IL-4 responses to protective Th1 response 10.

*TSA* gene is 600 *bp* and located on the chromosome 15 (22 *kDa*). Protein of TSA is expressed in promastigote and amastigote of *Leishmania* species 1,13–15.

LACK vaccine requires adjuvant of IL-12 to exert its protective efficacy against *Leishmania* 16.

IL-12 can redirect early Th2 response to *Leishmania* in murine, and it also can rapidly enhance the IL-4 production which is initiated basically from the Th2 cells maturation and development resistance against *Leishmania* parasite. Therefore, IL-12 is required for resistance to *L. major* 17.

Studies revealed a high protection in BALB/c model against *L. major* after DNA vaccine using a cocktail of plasmids 18. Given the recent investigations, murine model was protected against *L. major* due to pre-immunization with a cocktail of plasmids encoding to LACK, TSA and LmSTI1 antigens. This protection is commonly provided by the synergistic effect of humoral and cellular immunity 18–20.

Based on the literature, this study aimed at exploring the possibility of elevating the immune response by adding *IL-12* genetic adjuvant to the DNA cocktail containing plasmids encoding the genes of *L. major* and examining its immune response as well as protective efficacy in comparison with single-gene vaccine (pcLACK) and control groups (PBS, pcDNA3, pCAG GS-IL12).

## Materials and Methods

### Ethical statement

This project was approved by the Ethical Committee of School of Medical Sciences of Tarbiat Modares University and also by the Medical Ethics Committee in 2010.

### Mice

BALB/c mice were bought from Iran’s Razi Serum and Vaccine Production Research Institute. All mice were maintained under specific-pathogen-free conditions. Female mice aged between six to eight weeks were included in the survey.

### Parasites

Tarbiat Modares University Parasitology Department provided MHRO/IR/75/ER of *L. major*. Promastigotes were grown at 26°*C* in RPMI1640 medium (Sigma®) complemented with 10% heat- inactivated Fetal Calf Serum (FCS) (Gibco®), 100 *U/ml* of penicillin and 100 *μg/ml* of streptomycin. Promastigotes were collected at the stationary growth phase by centrifugation and finally washed three times with PBS.

### Construction of recombinant plasmids

The following *L. major* genes were chosen as DNA vaccine candidates: *LACK*, *TSA*. These genes were sub-cloned to the eukaryotic expression vector PcDNA3 by following standard molecular biology techniques. The single-gene expression plasmid of pcDNA3-LACK that encodes the LACK (939 *bp*, 36 *kDa*) antigen was constructed by our laboratory. Forward and reverse primers were designed as follows: forward primer introduced Hind III recognition site, underlined 5′**-**ATT AAG CTT ATG AAC TAC GAG GGT CAC CTG AAG GG-3′ and reverse primer introduced EcoRI recognition site underlined 5′-TTA GAA TTC TTA CTC GGC GTC GGA GAT −3′ 21.

The gene expression plasmid pcDNA3-TSA (pc TSA), encoding TSA (600 *bp*, 22 *kDa*) antigen was produced at Tarbiat Modares University, Iran 18.

To confirm the gene expression, six-histidine tag (6-His tag) sequence was designed in the forward primer. The designed forward and reverse primers were as follows: forward primer introduced Hind III recognition site underlined 5′-CAA TTA AA GCT TAT ATG CAT CAC CAT CAC CAT CAC ATG TCC TGC GGT AAC GCC AAG-3′, and reverse primer introduced EcoRI recognition site underlined 5′-CAT GGA ATT CTT ACT GCT TGC TGA AGT ATCC-3′.

Recombinant IL-12 plasmid contains a part of the gene expressing IL-12 which was constructed in the form of pCAGGS-IL-12 that was kindly donated by Dr. Masanori Matsui (University of Tokyo, Japan). All the recombinant plasmids were transformed into the TG1 *E. coli* strain.

### Plasmid purification

The plasmid extraction kit (Qiagen Germany) was used for plasmid purification according to the manufacturer’s recommendations. Then, plasmids concentration was measured by spectrophotometry at 260 and 280 *nm*. The 260:280 absorption ratio was between 1.8 and 2.0.

### Antigen preparation

Soluble *Leishmania* antigen was prepared from stationary phase promastigotes of *L. major* after a few passages in Schneider’s medium. About 2×10^6^ of *L. major* promastigotes were washed five times in cold sterile PBS containing 1 *mM* phenylmethylsulphonyl fluoride (PMSF). *L. major* antigen was prepared by freezing and thawing method. Concentration of antigen was measured by Bradford method. Prior to the usage, the prepared antigen was frozen at −20°*C*.

### Classification of mice

Initially, mice were grouped. The name and number of mice in each group are shown in table 1. Treatment groups were as follows: four control groups including PBS, pc-DNA3, pCAGGS-IL-12, and pCAGGS-IL-12+pcDNA3 and two experimental groups including pcLACK, pcLACK+pcTSA+pCAGGS-IL12.

**Table 1.** Classification of mice according to the injection substances

**Groups under study according to the injection substance**	**Total number of mice**	**Number of mice of immunological evaluations (no challenging)**	**Number of mice of immunological evaluations after challenging**	**Number of mice of survival time, diameter of lesion and weight evaluations after challenging**
**PBS**	15	5	5	5
**pcDNA3**	15	5	5	5
**pCAGGS-IL12**	15	5	5	5
**pCAGGS-IL12 pcDNA3+**	15	5	5	5
**pcLACK**	24	8	8	8
**pcLACK+ pcTSA+pCAGGS-IL12**	24	8	8	8

### Immunization and challenge of mice

Mice were immunized three times at a 3 week interval with 100 *μg* of plasmid DNA suspended in 100 *μl* sterile PBS, 50 *μl* in each thigh skeletal muscle.

Three weeks after the last immunization, the mice of each group (immunized and control mice) were challenged at the base of tail by the intradermal (*I.D*) route with 2×10^6^ promastigotes of *L. major* (strain MHRO/IR/75/ER). The lesions appeared at the second week in the control groups and the third week in the immunized groups. Thereafter, measuring the diameter of lesion (n=five per group) was monitored weekly at the site of inoculation by a Vernier caliper. Then, animals were sacrificed, and the spleens and serum samples were harvested for immunological analysis.

### Evaluation of cellular immunity

To evaluate the cellular immunity of mice, the level of total IL-4 and IFN-γ against *L. major* was tested by the two samples using the ELISA method (Farnandez-Botran, 2001). Seven weeks after the final injection, splenocyte suspensions were prepared from immunized mice as follows: the spleens were removed under sterile conditions and then crushed in a 6 *cm* sterile plate containing 5 *ml* of cold sterile PBS solution (4°*C*, the suspension was centrifuged for 10 *min* at 300×*g*, 4°*C*). Afterwards, lymphocytes were extracted from the spleen cultured in RPMI 1640 (Gibco, BRL, Maryland, USA), and 20% FCS (Gibco, BRL, Maryland, USA) in 24-well plates (Nalge-Nunc International, Rochester, NY, USA) at 2×10^6^ *cells/ml*. Then, they were allowed to multiply for 72 *hr* in the medium alone (control group), in the presence of SLA with concentration of 40 *lg/ml* (Sigma-Aldrich, Deisenhofen, Germany) incubated as mitogen at 37°*C* and 5% CO_2_. After collecting cell supernatant, a DuoSet ELISA Development Kit (R&D) was used to evaluate the presence of cytokines IL-4 and IFN-γ in the cellular suspension of the spleen lymphocytes of mice immunized with the aforementioned plasmids. The materials and solutions were prepared according to the guidelines of the kit, and then, the standard curve was drawn according to the current standard, and samples were finally evaluated.

### Evaluation of humoral immunity

The sera of mice were collected three weeks after the last vaccination and seven weeks after the challenge. Prior to usage, sera were kept at −20°*C*. To detect the levels of anti-*L. major* Ig G, the sera were tested with ELISA kits. The 96 wells-plates were coated at 4°*C* overnight with soluble *L. major* antigens at concentration of 10 *μg/ml* prepared in 100 *mM* carbonate-bicarbonate buffer (pH=9.6). Blocking was carried out with 5% dried skimmed milk in PBS (pH=7.2) for 2 *hr* at the room temperature. After washing with PBS containing 0.05% Tween 20 (Merck KGaA, Darmstadt, Germany) (PBST20), the sera were diluted 1/100 in 5% dried skimmed milk-PBST20 (100 *lL* per well) and incubated for 2 *hr* at 37°*C*. After washing, the bound antibodies were detected by incubation for 2 *hr* at 37°*C* with anti-mouse IgG conjugated with peroxidase (DA-KO, Denmark) at 1/2000 dilution in 5% dried skimmed milk-PBST20. Peroxidase activity was revealed by adding 100 *μl* per well of tetra methyl benzidine (Sigma-Aldrich) in dark. The reaction was stopped after adding 100 *μl* of sulfuric acid and Optical Density (OD) was read at 450 *nm* by the ELISA micro plate reader (Bio-Rad, USA) 22.

### Statistical analysis

Statistical comparisons between the experimental groups were carried out with an Analysis of variance (ANOVA) and Tukey test. Statistical analysis of survival time was applied with Kaplan-Meier (Log-Rank) test for five mice per each group. The level of significance was considered as p-value of less than 0.05.

## Results

### Evaluation of survival time in immunized and control mice after challenge

Results indicated that the survival rate in vaccinated group was significantly longer than the control groups (p<0.05). In group immunized with vaccine containing pcLACK, the survival rate increased more than the one with pcLACK+pcTSA+pCAGGS-IL12 and control groups (p<0.05). Mortality in the pCAGGS-IL12, pC-AGGS-IL-12+pcDNA3 control groups started respectively in six and seven weeks after the challenge, and all mice of control groups died in 13 weeks.

**Figure 1.**
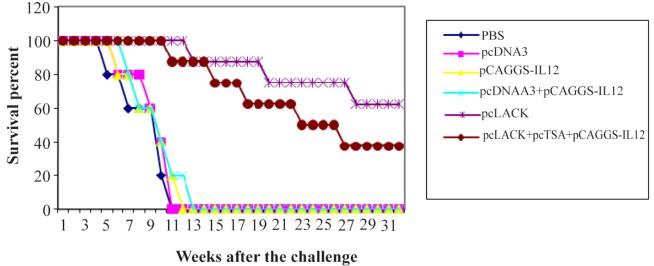
Survival of immunized BALB/c mice after challenge with 2×10^6^ promastigotes of *L. major*, four weeks after the last immunization.

But mortality in groups immunized with vaccine pc-LACK+pcTSA+pCAGGS-IL12, pcLACK began accordingly in 11, 13 weeks after the challenge of mice with *L. major*. Survival rate in the pcLACK group was 62.5% at week 32, while it was 37.5% in the pcLACK+pcTSA+pCAGGS-IL12 group These results state that the survival time in pcLACK group was significantly longer than the group vaccinated with pc-LACK+pcTSA+pCAGGS-IL12 (p<0.05) (Figure 1).

### Measurement of lesion size in immunized and control groups

**Figure 2.**
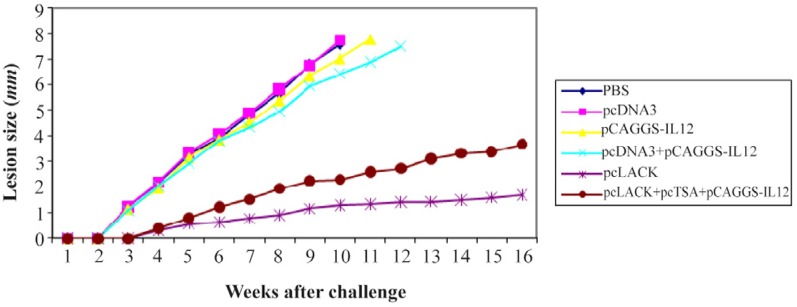
The lesion size of immunized BALB/c after the challenge with 2×10^6^ promastigotes of *L. major.*

The mean size of leisons was measured in immunized groups during 16 weeks after the challenge with 2×10^6^ *L. major* promastigotes. Lesions with mean size of 1.2 *mm* were observed three weeks after the challenge in the control groups, while, the mean size of lesions in the groups immunized with pcLACK+pc-TSA+pCAGGS-IL12, pcLACK 0.35 *mm* was observed four weeks after the challenge. Results indicated that the mean size of lesions in vaccinated group was meaningfully different from the control groups after the challenge (p<0.05) (Figure 2).

### Evaluation of number of parasites in the spleen cells of immunized mice after the challenge

**Figure 3.**
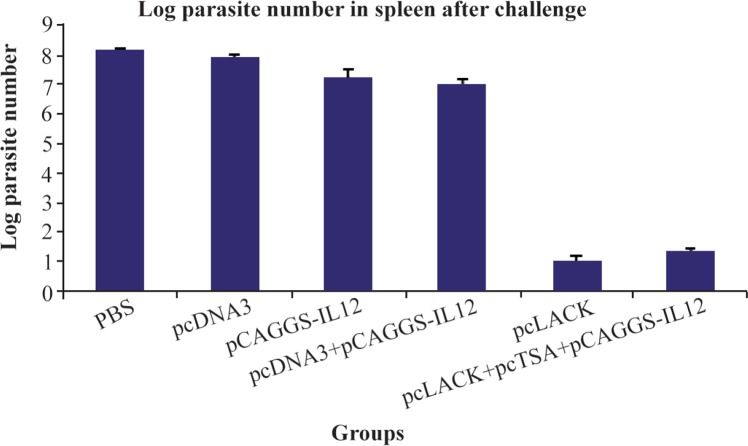
The logarithmic number of the parasites in the spleen cells of vaccinated mice and control groups after the challenge with *L. major* promastigotes.

The spleen of immunized mice after seven weeks of challenge with 2×10^6^ *L. major* promastigotes was removed in sterile conditions. The immunized mice had significantly lower parasite loads compared to the control mice (p<0.05). The group vaccinated with pc-LACK+ showed the most amount of reduction in parasite burden in comparison with pcLACK+pcTSA+pC AGGS-IL12 group (Figure 3).

### Evaluation of the level of IgG (anti-L. major) in the immunized mice

**Figure 4.**
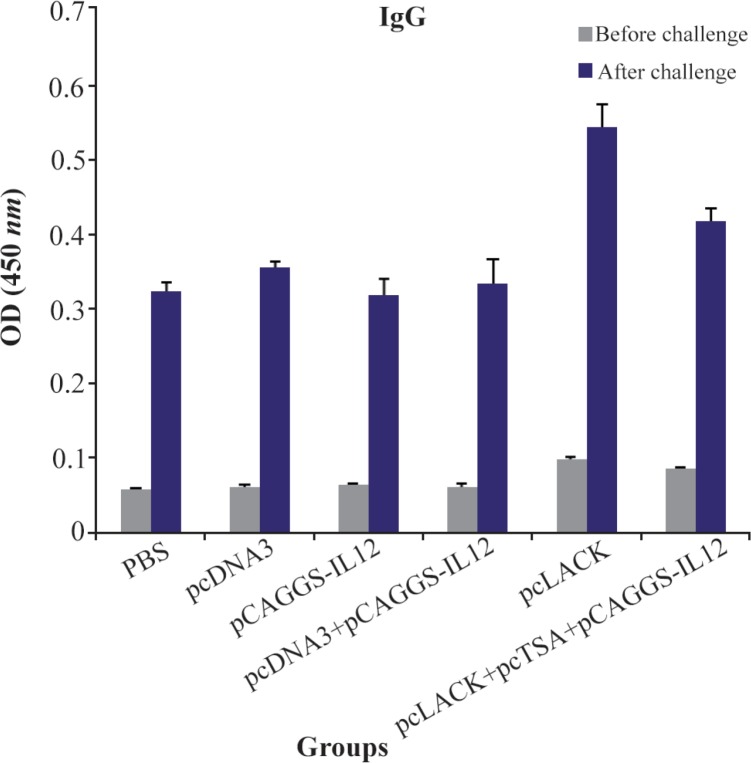
Analysis of the total IgG antibodies in pooled sera of mice. The sera were collected three weeks after the last vaccination and seven weeks after the challenge. Results are expressed as mean of the OD450 *nm*±SD of the samples.

To study humoral immunity, the level of total IgG against *L. major* was measured on the sera using the ELISA method. Serum samples were collected from the immunized and control mice three weeks after the final booster injection, and also seven weeks after challenge infection from five mice of each group in the control and eight mice in the immunized group by random selection. Comparison of ODs obtained by the total IgG among the different groups with Mann-Whitney test showed that anti-*L. major* IgG values increased significantly in the vaccinated groups compared to the control groups in pre and post challenge (p<0.05). The difference between pcLACK vaccine and pc LACK+pcTSA+pCAGGS-IL12 was significant in pre- and post-challenge as well (p<0.05) (Figure 4).

### Measurement of the cytokine production in the immunized mice

**Figure 5.**
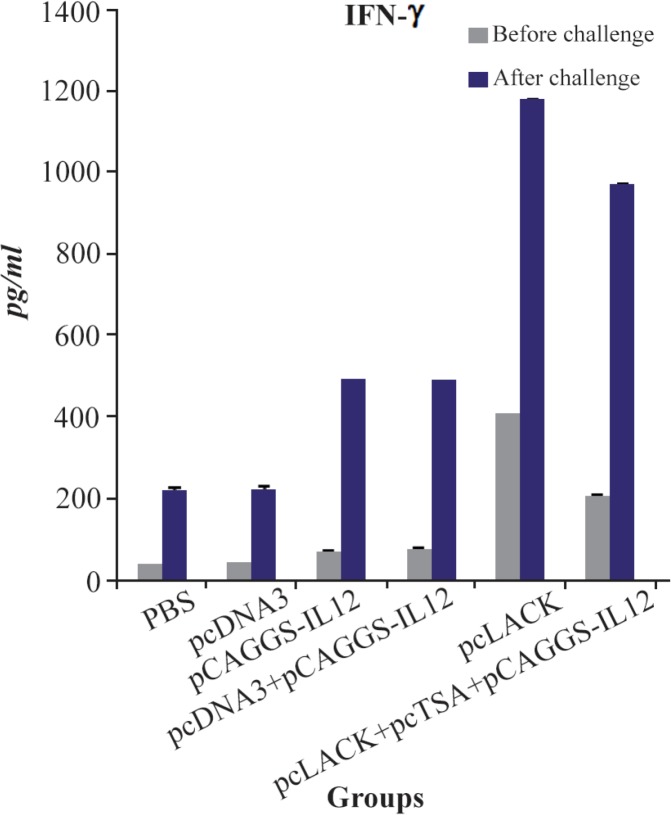
IFN-ɣ productions of *L. major* antigen-stimulated spleno-cytes from vaccinated and control mice three weeks after the last vaccination and seven weeks after challenge infection using the ELISA method. Results are expressed as means of the OD450 *nm*±SD.

To evaluate the cellular immune response in the immunized mice, lymphocyte suspensions were prepared from the spleen of the immunized and control mice three week after the final booster injection and seven weeks after challenge infection. The mean level of IFN-γ between the immunized and control groups was significantly different (p<0.05). Also the level of IFN-γ in mice immunized with pc-LACK was significantly higher compared with those immunized with pc-LACK+pcTSA+pCAGGS-IL12 (Figure 5).

**Figure 6.**
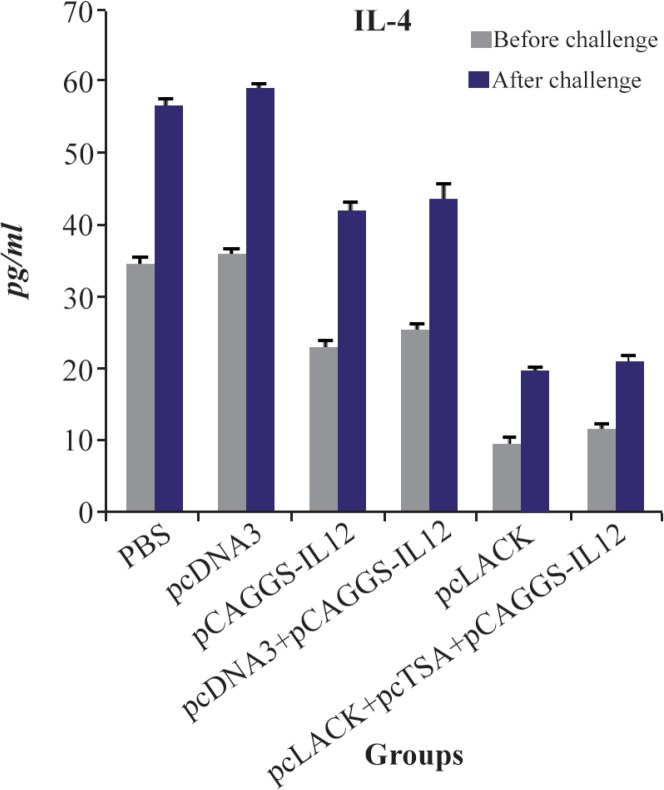
IL-4 productions of *L. major* antigen-stimulated spleno-cytes from vaccinated and control mice three weeks after the last vaccination and seven weeks after challenge infection using the ELISA method.

The mean level of IL-4 produced by the spleen cells between the immunized and control groups was significantly different (p<0.05). But decreased level of IL-4 in the group vaccinated with the pc-LACK was higher than the group immunized with pcLACK+pcTSA+ pCAGGS-IL12 after and before the challenge; however, this difference was not significant (p<0.05) (Figure 6).

## Discussion

DNA vaccines can be useful for protection against leishmaniasis for developing naturally acquired immunity due to primary exposure to *L. major*. Low dose infection in the skin has recently been shown to be depended on CD4+ and CD8+ T cells 12,23,24.

A lot of progress has been achieved in the field of vaccine against leishmaniasis. *Leishmania* parasites have different antigens reflecting the complexity of the immune responses against the parasites.

DNA vaccine cocktail is an important strategy for developing the DNA vaccines 25. Studies showed that recombinant genetic adjuvants, IL-12, are required in the development of cellular immune response. The expression of biologically active IL-12 as immune regulation can protect against the intracellular parasites, *L. major* 26,27.

*LACK* and *TSA* genes of *Leishmania* are candidates of DNA vaccine for *L. major*. Immunization of BALB/c mice with DNA vaccine cocktail of pcTSA+pcLACK showed strong cellular and humoral immune responses against *L. major* 18,28.

At present, the ability of pcTSA+pcLACK with pCAGGS-IL-12 genetic adjuvant to elicit protective immunity in DNA vaccine strategy was investigated. Immunization of BALB/c mice with pcTSA+pcLACK +pCAGGS-IL-12 cited the development of cellular immune responses against *L. major*, while, current findings indicated that immunization of mice with single pcLACK stimulated cellular immune responses which are stronger than vaccination with pcTSA+pc-LACK+pCAGGS-IL-12. The level of IFN-ɣ was enhanced strongly in mice immunized with single pc-LACK, compared with mice immunized with pcTSA+ pcLACK+pCAGGS-IL-12. This result shows that pc-LACK, IL-12 develops Th1 cytokine responses and is effective in treatment of CL 13,18,26.

Detection of total IgG indicated that IgG values against *Leishmania* were increased markedly in the pcTSA+pcLACK+pCAGGS-IL-12 group compared to the control groups. Anti-leishmania IgG values in the group immunized with single pc-LACK were stronger than the group that received pcTSA+pcLACK+pC-AGGS-IL-12; however, there was no statistically significant difference between the two groups.

The current results presented that mice immunized with single pcLACK had a longer average survival time than the pcTSA+pcLACK+pCAGGS-IL-12 group. According to the present results, pc-LACK seems to play an important role in the efficacy of Th1 immune response. Sa′nchez-Sampedro *et al* showed that the ex-pression of LACK protein in a DNA prime/MVA boost regimen can lead to high protective efficacy and a protective Th1 immune response 12,29,30.

## Conclusion

It is expected that the use of DNA cocktail with pCAGGS-IL-12 leads to IFN-γ increase and IL-4 decrease, respectively. But, there was no significant difference between groups that received pCAGGS-IL-12 or the ones who did not receive pCAGGS-IL-12. So, it was not possible that the three genes stimulated equally the immune response in a short time.

Our investigation provided basic information for researches in the use of DNA vaccines combined with recombinant plasmid and adjuvant of cytokine in studies of protection against *L. major*.
